# A simple and robust method for pre-wetting poly (lactic-co-glycolic) acid microspheres

**DOI:** 10.1177/0885328215577297

**Published:** 2015-08

**Authors:** Bernice Wright, Nina Parmar, Laurent Bozec, Sebastian D Aguayo, Richard M Day

**Affiliations:** 1 Applied Biomedical Engineering Group, Division of Medicine, University College London; 2 Division Biomaterials and Tissue Engineering, UCL Eastman Dental Institute, University College London

**Keywords:** Poly (lactic-co-glycolic) acid microspheres, thermally induced phase separation, thermally induced phase separation microspheres, serum protein adsorption, human skeletal myoblasts and thermally induced phase separation microspheres, poly (lactic-co-glycolic) acid microsphere surface topography, wetting poly (lactic-co-glycolic) acid microspheres

## Abstract

Poly (lactic-co-glycolic) acid microspheres are amenable to a number of biomedical procedures that support delivery of cells, drugs, peptides or genes. Hydrophilisation or wetting of poly (lactic-co-glycolic) acid are an important pre-requisites for attachment of cells and can be achieved via exposure to plasma oxygen or nitrogen, surface hydrolysis with NaOH or chloric acid, immersion in ethanol and water, or prolonged incubation in phosphate buffered saline or cell culture medium. The aim of this study is to develop a simple method for wetting poly (lactic-co-glycolic) acid microspheres for cell delivery applications. A one-step ethanol immersion process that involved addition of serum-supplemented medium and ethanol to PLGA microspheres over 30 min–24 h is described in the present study. This protocol presents a more efficient methodology than conventional two-step wetting procedures. Attachment of human skeletal myoblasts to poly (lactic-co-glycolic) acid microspheres was dependent on extent of wetting, changes in surface topography mediated by ethanol pre-wetting and serum protein adsorption. Ethanol, at 70% (v/v) and 100%, facilitated similar levels of wetting. Wetting with 35% (v/v) ethanol was only achieved after 24 h. Pre-wetting (over 3 h) with 70% (v/v) ethanol allowed significantly greater (*p* ≤ 0.01) serum protein adsorption to microspheres than wetting with 35% (v/v) ethanol. On serum protein-loaded microspheres, greater numbers of myoblasts attached to constructs wetted with 70% ethanol than those partially wetted with 35% (v/v) ethanol. Microspheres treated with 70% (v/v) ethanol presented a more rugose surface than those treated with 35% (v/v) ethanol, indicating that more efficient myoblast adhesion to the former may be at least partially attributed to differences in surface structure. We conclude that our novel protocol for pre-wetting poly (lactic-co-glycolic) acid microspheres that incorporates biochemical and structural features into this biomaterial can facilitate myoblast delivery for use in clinical settings.

## Introduction

**Figure 1.**
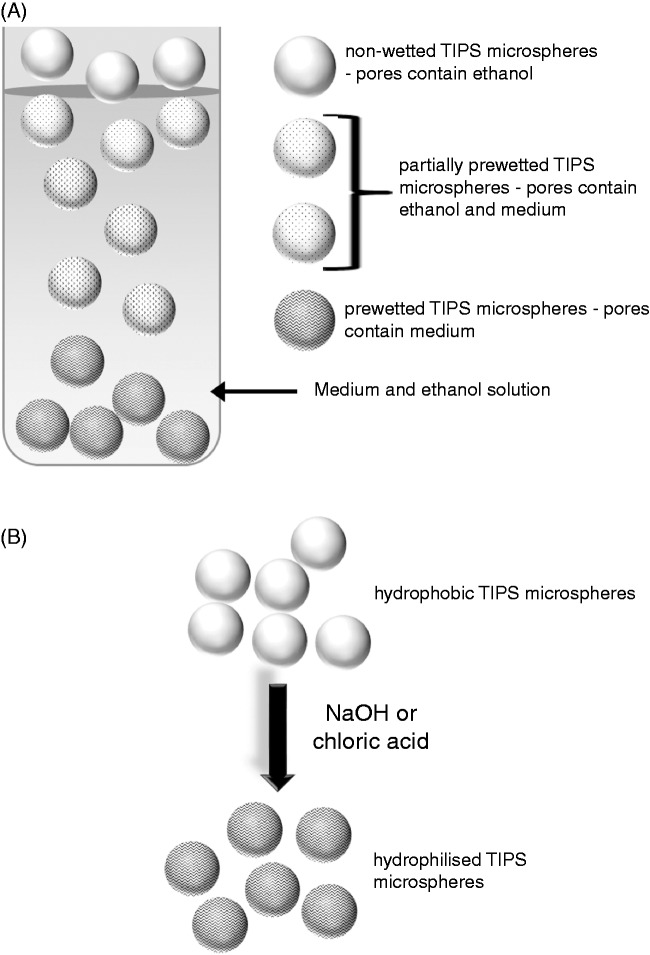
Wetting and hydrophilisation of hydrophobic polymers. Wetting (a) is a simple procedure that is achieved with diluted ethanol. The ethanol is exchanged for culture medium until pore spaces in the polymer are filled with medium, allowing the polymer to become submerged. Hydrophilisation (b) involves hydrolysis of a polymer by a strong alkali (e.g. NaOH) or acid (e.g. chloric acid solution) to create hydroxyl or carboxyl groups on the polymer surface.

Poly (lactic-co-glycolic) acid (PLGA) is a synthetic, biocompatible copolymer that is commonly used as a cell^1^ and protein^2^ delivery scaffold. Due to intrinsic hydrophobic properties, PLGA polymers require pre-treatment to wet or hydrophilise their surface and thereby allow cell attachment. A number of approaches including ethanol immersion,^3,4^ chemical modification with alkaline solutions (e.g. NaOH)^5^ and plasma oxygen^6,7^ are demonstrated as viable techniques for improving the hydrophilicity, pre-wetting or hydrophilisation of PLGA. The ethanol-mediated pre-wetting method involves the exchange of ethanol for culture medium in microsphere pores to eventually submerge these constructs and enable contact with cells, whereas hydrophilisation involves hydrolysis of the microsphere surface with strong acids or alkalis (Figure 1). For laboratory and clinical application of PLGA microspheres, the simple, practical, ethanol immersion method for pre-wetting PLGA is the best approach. The aim of the current study is to describe optimal conditions for ethanol-dependent wetting of PLGA microspheres that support efficient loading of human skeletal myoblasts.

In the present study, PLGA microspheres fabricated with the thermally-induced phase separation (TIPS) technology were employed. This specialized technique allows formation of monodisperse, highly porous microspheres via rapid removal of thermal energy in a liquid nitrogen bath.^8^ The key advantages of TIPS PLGA microspheres include their open pore structure that facilitates controlled, sustained release of small-molecule drugs (e.g. antibiotics)^9^ and antibodies,^2^ an external surface that supports adhesion and proliferation of cells^1^ and a predictable degradation profile.^10^ Moreover, TIPS microcarriers require less initial polymer material to be formed than the more commonly used solid PLGA microspheres, due to their intrinsic porosity. We have found that *in vivo*, TIPS microspheres can integrate into tissue and are infiltrated by inflammatory cells (unpublished data). The present study will indicate wetting conditions for these scaffolds that will allow optimal loading with human skeletal myoblasts.

Wetting or hydrophilisation of hydrophobic polymers is an essential pre-requisite for cell attachment.^11–14^ A previous study demonstrated that poly (glycolic acid) fibre meshes pre-wetted with medium supported attachment of murine hepatocytes.^15^ Moderately hydrophilic surfaces rather than those that were hydrophobic or completely hydrophilic were reported to support endothelial cell adhesion on polyethylene (PE) glycol films, and the strength of adhesion was dependent on maintaining partial hydrophilisation.^16^ Moderate wetting of hydrophobic methacrylate polymers has been shown to allow efficient adhesion of endothelial cells, but greater numbers of cells adhered in the presence of serum compared to serum-free conditions.^14^ A related study showed that moderately wetted polymer surfaces exhibited a serum and cellular protein adsorption pattern that was favorable for attachment of endothelial cells.^13^

Further studies investigating wettability and cell adhesion focused on the use of the wettability gradient of polymers to study the link between protein adsorption and cell adhesion.^11,17^ These studies demonstrated that various cell types (Chinese hamster ovary cells, mouse embryo fibroblasts, bovine pulmonary artery cells) adhered, spread and grew in positions along the wettable gradient surface that were moderately hydrophilic on PE films.^11,17^ Chargeable functional group gradient surfaces on wetted PE sheets, where the surface density of grafted functional groups (carboxylic acid, sulphonate and amine groups) changed gradually along the sample length, supported the attachment and growth of Chinese hamster ovary cells.^17^

Human albumin adsorption to PE sheets was reported to decrease along the wettability gradient, therefore increasing along the hydrophobic regions of this polymer.^12^ Despite this study, human serum adsorption has been correlated to adhesion of human endothelial cells to moderately hydrophilic polymers,^13^ possibly due to preferential adsorption of serum proteins to hydrophilic regions.^11^

Structural modifications to PLGA due to polymer composition, method of manufacture and nanostructured surface features were previously shown to influence the binding of serum proteins as well as cells to this polymer. Pluronic® F-108 blended PLGA microfibrous scaffolds adsorbed significantly greater amounts of bovine serum albumin than unblended PLGA.^18^ PLGA microspheres fabricated via a water/oil/water (w/o/w) emulsion solvent evaporation method adsorbed greater levels of albumin, IgG, gelsolin and β2-Glycoprotein I than those prepared using a spray-drying technique. The latter technique, however, allowed greater adsorption of apolipoproteins, Ig light chains and IgD.^19^ PLGA scaffolds synthesized to present surface features ranging from micron to nanometer, allowed greater adhesion of endothelial and smooth muscle cells compared to unmodified PLGA.^20^ Similar topographical changes (submicron to nanometer surface features) to PLGA reduced fibronectin adsorption and platelet adhesion to these proteins on the polymer.^21^

In the present study, TIPS microspheres were wetted to various extents by exposure to different concentrations of ethanol. Serum protein adsorption to TIPS microspheres was shown to accelerate wetting. Human skeletal myoblast attachment to microspheres is influenced by both wetting and the amount of protein adsorbed to these scaffolds, with high amounts of serum protein enhancing the efficiency of cell attachment to less wetted microspheres. We attributed differences in cell attachment to changes in surface topography that demonstrated a more rugose surface structure for more wetted microspheres compared to those that were less wetted.

Taken together, the present study describes a protocol for the preparation of TIPS PLGA microspheres that can be used to facilitate cell delivery with these material scaffolds through dual modification of wetting and protein adsorption parameters.

## Materials and methods

### Materials

Nutrient mixture F-10 Ham, dexamethasone, antibiotics (penicillin/streptomycin), Trypan Blue dye solution, Tris-base, acetic acid, Trypsin/EDTA solution, sodium dodecyl sulphate (SDS), 2-mercaptoethanol, ammonium persulphate (AMPS), N,N,N′,N′-tetramethylethylenediamine (TEMED), silver nitrate, sodium carbonate, sodium acetate, ethanol, formaldehyde, ethylenediaminetetraacetic acid disodium dehydrate, bovine serum albumin (BSA), the Micro Lowry (Peterson’s Modification) total protein assay kit, bromophenol blue, glycerol and sodium thiosulphate were purchased from Sigma-Aldrich (Poole, UK). Industrial methylated spirits (IMS; 99% ethanol) was obtained from Barrettine Industrial Ltd. (Bristol, UK) and human fibroblast growth factor (FGF) basic was acquired from Peprotech (London, UK). Dulbecco’s modified eagle’s medium (DMEM)/Ham F-12 (1:1), fetal bovine serum (FBS) and the Cyquant® NF assay kit were from Life Technologies (Paisley, UK). Methanol and acetic acid were purchased from Thermo Scientific HyClone (Fisher Scientific: Leicestershire, UK) and acrylamide (30% acrylamide/Bis solution) was from BioRad (Hertfordshire, UK). The human skeletal myoblasts were from Lonza Biologics (Slough, UK) and the VectorShield mounting medium with 4′,6-diamidino-2-phenylindole (DAPI) was from Vector Laboratories Ltd. (Peterborough, UK). TIPS PLGA (Purasorb PDLG 7507, Purac Biomaterials, Gorinchem, The Netherlands) microspheres (75:25, inherent viscosity approximately 0.6 dl g^−1^) were prepared as previously described.^1,2^

### Methods

#### Pre-wetting PLGA TIPS microspheres

PLGA TIPS microspheres were fabricated^1,2^ using 75:25 poly(d,l-lactide-co-glycolide) (Purasorb PDLG 7507, Purac Biomaterials, Gorinchem, The Netherlands) that was dissolved in dimethylcarbonate (1:25 (w/v), Sigma–Aldrich, UK). Polymer solution was fed into Nisco Encapsulator Unit Var D (Nisco Engineering, Zurich, Switzerland) by a syringe pump (Pump 11, Harvard Apparatus, Kent, UK), connected via a silicone tube, at a constant rate of 3 mL min^−1^, with vibration frequency of the nozzle at 1.80 kHz and the amplitude of frequency at 100%. Liquid polymer droplets were ejected into a PE beaker containing 250 mL of liquid nitrogen, and frozen droplets were equilibrated in the liquid nitrogen, before samples were freeze dried (Edwards MicroModulyo freeze dryer, Thermo Fisher Scientific, Asheville, NC) for 24 h to allow the sublimation of residual dimethylcarbonate. TIPS microspheres were sieved to produce batches with diameters of 200–300 µm in diameter and scanning electron microscopy confirmed their spherical morphology.

PLGA TIPS microspheres were wetted with industrial methylated spirits (IMS) containing 99% ethanol. IMS was used instead of 100% ethanol as this solution allowed TIPS microspheres to be wetted in a similar manner as ethanol. In future studies where the pre-wetting protocol will be scaled up to a high throughput bioprocess, for large-scale commercial production of wetted TIPS microspheres, the use of IMS for pre-wetting TIPS microspheres will be replaced with ethanol. Dry microspheres were weighed into bijou tubes and 3 mL DMEM/F12 containing 0%, 2%, 10% or 20% FBS was pipetted over the polymer. IMS (600 µL) at 35% (v/v), 70% (v/v) or 100% was mixed into the medium by gentle pipetting. Microspheres were incubated in the medium/IMS solution for 30 min, 3 h and 24 h before the tube was photographed to image the sinking of the polymer from the surface of the solution as it became wetted. After the pre-wetting period, the microspheres were washed with sterile nanopure water – 3 mL and 1 mL of water were used for the first and second washes, respectively. For quantification of proteins adsorbed to the microsphere surface, the individual polymer preparations were suspended in 100 µL of water before the protein assay was performed. PLGA microspheres used for myoblasts attachment studies were prepared using Nutrient medium Ham F-10 instead of DMEM/F12 as the base medium.

#### Total protein estimations

The micro Lowry protein assay kit was used to measure the amount of serum proteins adsorbed to the surface of PLGA microspheres pre-wetted in the presence of FBS. BSA dissolved in nanopure water was used as a protein standard at 50, 100, 200, 300, 400, 500 and 1000 µg/mL concentrations. The Lowry reagent solution (250 µL) was added to 100 µL of each BSA standard, microsphere preparation (suspended in 100 µL nanopure water) or 100 µL water (blank). The solutions were mixed well by vortexing briefly at high speed and incubated at room temperature for 20 min. Folin and Ciocalteu’s phenol reagent working solution (125 µL) was added to each tube with rapid and immediate mixing and the colour was allowed to develop for 30 min. The solutions (100 µL) were transferred to a 96-well plate and absorbance was measured at a wavelength between 500 and 800 nm within 30 min.

#### One-dimensional gel electrophoresis

Sodium dodecyl sulphate – polyacrylamide gel electrophoresis (SDS-PAGE) was carried out in discontinuous vertical slab gels which contained a final concentration of 15% v/v acrylamide in the resolving gel and 4% (v/v) acrylamide in the stacking gel. The stacking gel contained stacking gel buffer (0.5 M Tris-base; pH 6.8), 30% (v/v) acrylamide, 10% (w/v) SDS, 0.05% (v/v) TEMED and 1.5% (w/v) AMPS. The resolving gel comprised resolving gel buffer (3 M Tris-base and 5 N HCl; pH 8.8), 30% (v/v) acrylamide, 10% (w/v) SDS, 0.08% (v/v) TEMED and 1.5% (w/v) AMPS.

#### Silver nitrate protein staining

Serum proteins and albumin were stained with silver nitrate. Briefly, proteins immobilized in 15% v/v acrylamide gels were fixed in a solution containing 40% (v/v) methanol, 10% (v/v) acetic acid and 50% (v/v) water. Gels were then washed three times for 5 min each time before they were treated with (5% (w/v) sodium thiosulphate, 30% (v/v/) ethanol and 68 mg/mL sodium acetate) for 30 min. The gel was then washed three times with deionised water for 10 min each time, and stained with 2.5% (w/v) silver nitrate for 20 min. Stained gels were washed twice with deionised water (2 min per wash, and the silver stain was developed using a solution of 2.5% (w/v) sodium carbonate and 0.04% (v/v) formaldehyde. When protein bands were visible, development of the stain was stopped using a 1.46% (w/v) EDTA-Na_22_H_2_O solution. The stained gels were washed with water and imaged using an Epson gel scanner. Gels were stored in 5% (v/v) acetic acid at 4℃.

#### Culture of human skeletal myoblasts

Human skeletal myoblasts (Lonza Biologics) were initially cultured to 70–80% confluency on tissue culture plastic in supplemented (FBS (20%), human FGF-basic (1 µg/mL), dexamethasone (100 µM) and antibiotics (penicillin/streptomycin) (1%)) Nutrient mixture F-10 Ham. Myoblasts were then detached from culture flasks using Trypsin/EDTA solution, counted using a haemocytometer and evaluated for live and dead cell proportions using the Trypan blue exclusion assay (the number of dead cells were negligible). Myoblasts (5 × 10^4^) were seeded onto PLGA TIPS microspheres (approximately 250 microspheres) pre-wetted with 35% or 70% IMS in the presence of 0%, 2% or 20% FBS. The cells and microspheres were cultured in 100 µL supplemented Nutrient mixture F-10 Ham in a 96-well plate for 6 h. The plate was shaken for 10 s every hour at approximately 500 r/min to lift both the myoblasts and the microspheres, to allow the cells to maintain contact with the polymer. After the culture period, the cells that remained unattached to the polymer were washed away with sterile phosphate buffered saline (PBS).

#### Cell quantification using the Cyquant® NF assay

The number of myoblasts attached to TIPS microspheres pre-wetted under various conditions was measured using the Cyquant® NF assay that determines levels of cellular DNA via fluorescent dye binding. The dye reagent (50 µL) was incubated with PLGA/myoblasts constructs for 1 h at 37℃. Fluorescence was excited at 485 nm and emitted at 530 nm and the signals generated were correlated with that from a standard curve of known cell numbers to determine the number of cells adhered to microspheres.

#### Fluorescence microscopy

The PLGA/myoblast constructs were fixed with 4% (w/v) for 1 h and mounted with VectorShield containing DAPI on a glass slide. A coverslip was placed over the glass slide prior to analysis. Fluorescence was excited at 358 nm and emitted at 461–488 nm and three-dimensional representations of nuclei from myoblasts attached to the surface of PLGA microspheres were constructed by the generation of layers in the z dimension which were compiled into z-stacks.

#### Atomic force microscopy

Pre-wetted TIPS microspheres were mounted on sterile glass slides using super glue and imaged under deionized water in intermittent contact (liquid) mode employing a JPK NanoWizard II AFM (JPK Instruments, Germany) mounted on an Olympus IX71 inverted microscope. MSNL-10 cantilevers (Bruker, Santa Barbara, USA), tuned to a drive frequency of ∼46 kHz (nominal resonant frequency 25–50 kHz), were employed with a constant line rate of 0.5 Hz. Gain parameters and set point were adjusted according to each sample. Thermal resonance calibration yielded a cantilever spring constant of 0.11 N/m (nominal value 0.1 N/m). After focusing on an area of interest, images with different scan sizes of 5 × 5 µm were obtained at random sites of each sample and processed with the Gwyddion 2.37 SPM software.

#### Statistical analysis

Unpaired *t*-tests were performed using Microsoft Excel to determine the statistical significance of quantified data. Results are presented as the mean of three individual experiments with standard error of mean (SEM) and *p* value ≤ 0.05 considered significant.

## Results

### Wetting of PLGA microspheres is enhanced with exposure to increasing concentrations of ethanol

To wet PLGA microspheres we performed a one-step ethanol immersion method. Ethanol was added to TIPS microspheres suspended in serum-supplemented cell culture medium. The sinking of microspheres in medium was used as an indicator of wetting, as previously described.^22^

**Figure 2.**
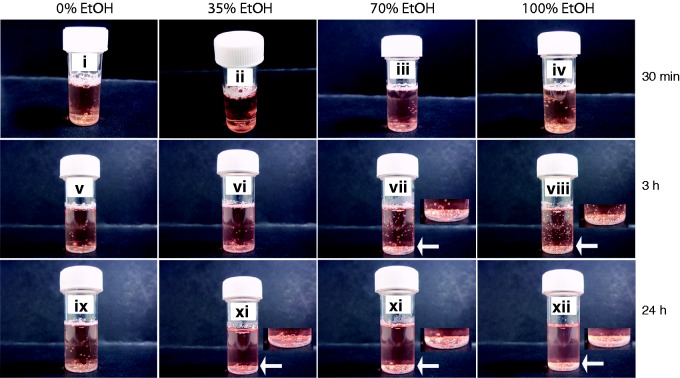
Wetting of TIPS PLGA microspheres by ethanol is dose-dependent. PLGA microspheres were suspended in medium containing 20% FBS and treated with 35% (v/v), 70% (v/v) or 100% ethanol (EtOH), for 30 min, 3 h and 24 h. Arrows and image insets show detailed images of submerged microspheres. Images represent three individual experiments.

Microspheres were wetted with 35% (v/v), 70% (v/v) and 100% industrial methylated spirits (containing 99% ethanol) over 30 min, 3 h and 24 h time periods, under standard culture conditions (37℃, 5% CO_2_, 95% humidity). We observed that microsphere pre-wetting was dependent on ethanol concentration and the length (30 min, 3 h and 24 h) of the wetting procedure. Microspheres exposed to 70% (v/v) and 100% ethanol became immersed in medium (containing 20% FBS) after 3 h, but those exposed to 35% (v/v) ethanol required 24 h before they were completely submerged (Figure 2).

These data demonstrated that PLGA microsphere wetting can be modified to various extents by altering ethanol concentrations and exposure times with this solvent. To our knowledge, our study is the first to show that dose- and time-dependent exposure to ethanol can be used to wet PLGA TIPS microspheres.

### Serum protein adsorption to PLGA microspheres accelerates wetting

Serum protein adsorption was previously linked to hydrophilisation of hydrophobic polymers.^11,13^ We, therefore, investigated the correlation between serum protein adsorption and wetness of PLGA TIPS microspheres. Microspheres were incubated with 35% (v/v) or 70% (v/v) ethanol in medium containing 2%, 10% and 20% serum (FBS).

**Figure 3.**
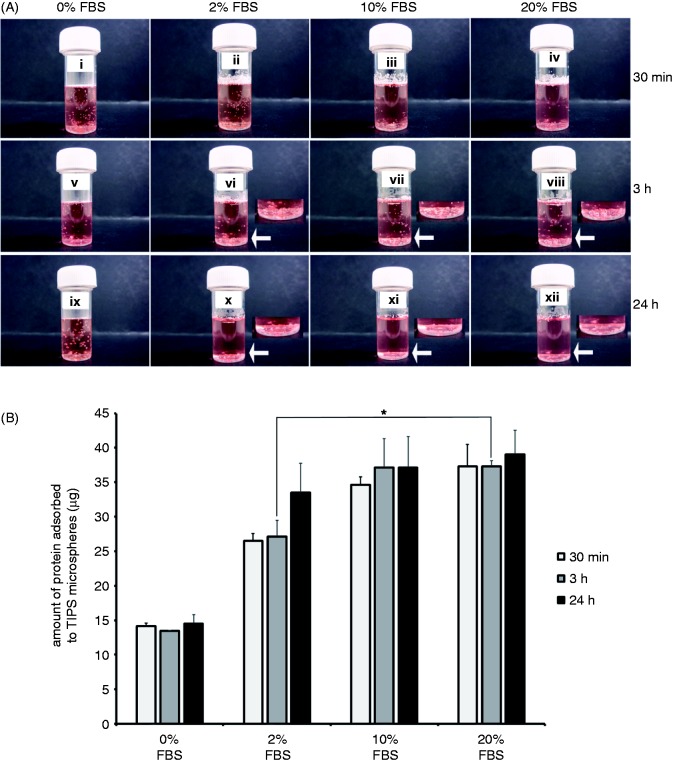
Serum proteins adsorb to wetted TIPS microspheres in a dose-dependent manner. PLGA microspheres were suspended in medium containing 0%, 2%, 10% or 20% FBS and treated 70% (v/v) ethanol (EtOH), for 30 min, 3 h and 24 h (a). Arrows and image insets show detailed images of submerged microspheres. The total amount of serum protein adsorbed to microspheres was measured after each incubation period (b). Data points represent the mean (n = 3 ± SEM) amount of total protein. *p ≤ 0.05 indicates differences between wetting conditions. Images represent three individual experiments.

**Figure 4.**
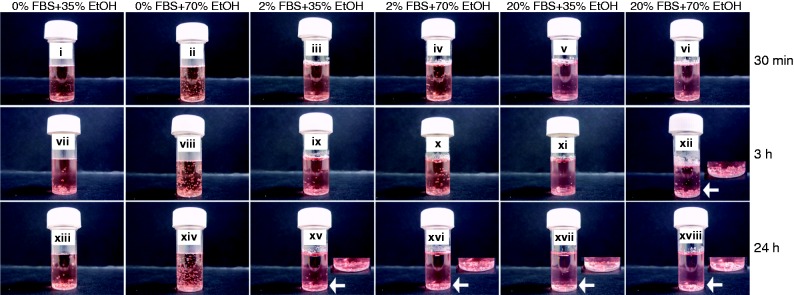
Serum proteins accelerate ethanol-mediated wetting of PLGA microspheres. PLGA microspheres were suspended in medium containing 0%, 2% or 20% FBS and treated with 35% (v/v) or 70% (v/v) ethanol (EtOH), for 30 min, 3 h and 24 h. Arrows and image insets show detailed images of submerged microspheres. Images represent three individual experiments.

Microspheres became completely submerged in 20% (v/v) and 10% (v/v) FBS preparations (with 70% (v/v) ethanol for wetting) after 3 h, but those exposed to 2% (v/v) FBS became immersed in medium after 24 h (Figure 3a.). After 3 h microspheres exposed to 20% (v/v) or 10% (v/v) FBS adsorbed similar amounts of protein (approximately 24 µg total protein), and a significantly lower (*p* ≤ 0.05) amount of protein (approximately 14 µg total protein) was adsorbed to those exposed to 2% (v/v) FBS (Figure 3b). Under conditions applying 35% (v/v) ethanol, that allowed only partial wetting of microspheres, microspheres did not sink after 3 h (Figure 4). Instead, after 24 h a proportion of microspheres treated with 2% FBS and 35% or 70% ethanol became submerged, but the majority of those treated with 20% FBS including 35% or 70% ethanol sank in medium (Figure 4).

**Figure 5.**
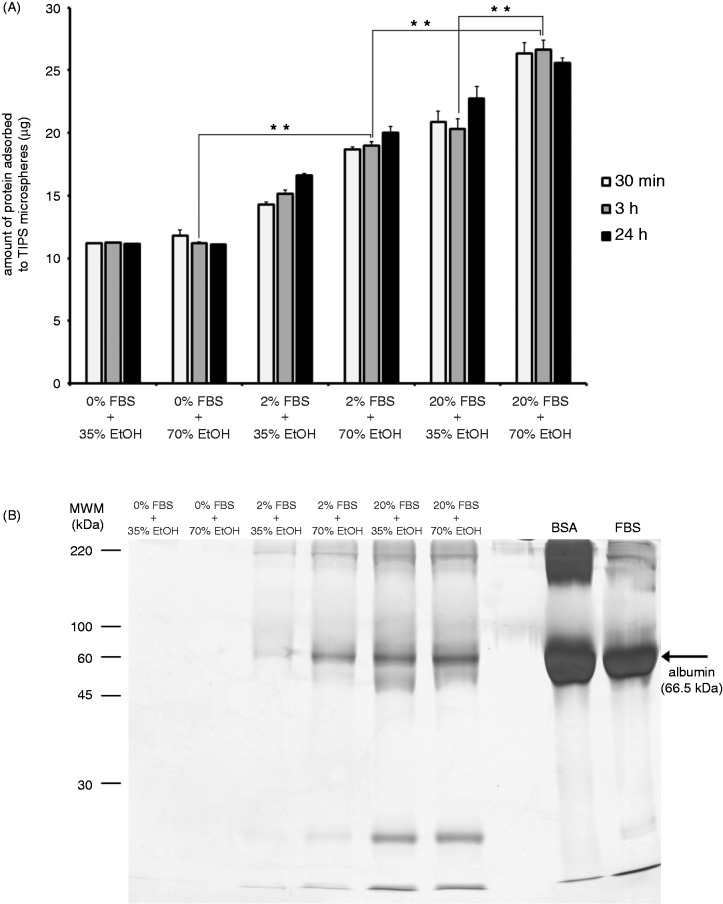
The adsorption of serum proteins to PLGA microspheres is dependent on the extent of pre-wetting. PLGA microspheres were suspended in medium containing 0%, 2% or 20% FBS and treated with 35% (v/v) or 70% (v/v) ethanol (EtOH), for 30 min, 3 h and 24 h. The total amount of serum protein adsorbed to microspheres was measured after each incubation period (a). Serum and EtOH-treated microspheres were heated (95℃) in Laemmli buffer containing the reducing agent β-mercaptoethanol, proteins were separated using 15% acrylamide gels and stained using silver nitrate (b). Bovine serum albumin (BSA) and fetal bovine serum (FBS) were included as external controls. Data points represent the mean (n = 3 ± SEM) amount of total protein. *p ≤ 0.05 indicate differences between wetting conditions. Gel represents three individual experiments from three different sets of microsphere-protein adsorption experiments.

Microspheres treated with 2% FBS + 70% ethanol and those treated with 20% FBS + 35% ethanol adsorbed similar amounts of serum proteins (Figure 5a). By contrast after a 3-h incubation, microspheres treated with 20% FBS + 70% ethanol adsorbed significantly greater (*p* ≤ 0.01) amounts of serum protein than those treated with 20% FBS + 35% ethanol, suggesting that the increased wetness of microspheres conferred by 70% (v/v) ethanol enhanced protein adsorption. The amount of protein adsorbed to microspheres wetted with 70% (v/v) ethanol increased as levels of FBS were raised. 0% FBS + 70% ethanol preparations adsorbed significantly less (*p* ≤ 0.01) amounts of protein than 2% FBS + 70% ethanol microspheres, which adsorbed significantly less (*p* ≤ 0.01) protein than 20% FBS + 70% ethanol microspheres. In preparations including 2% FBS, levels of albumin adsorbed to microspheres increased as the wetness of the polymers was enhanced, i.e. 70% ethanol allowed more albumin to adsorb than 35% ethanol (Figure 5a and b). Microspheres incubated with 20% FBS adsorbed similar levels of albumin whether they were partially wetted (35% ethanol) or completely wetted (70% ethanol), suggesting that the polymer may be saturated with protein when incubated with 20% FBS.

These data indicated that an additional level of control for wetting PLGA microspheres can involve adsorption of serum proteins.

### Human skeletal myoblast attachment to PLGA microspheres is dependent on the extent of wetting and surface topography

**Figure 6.**
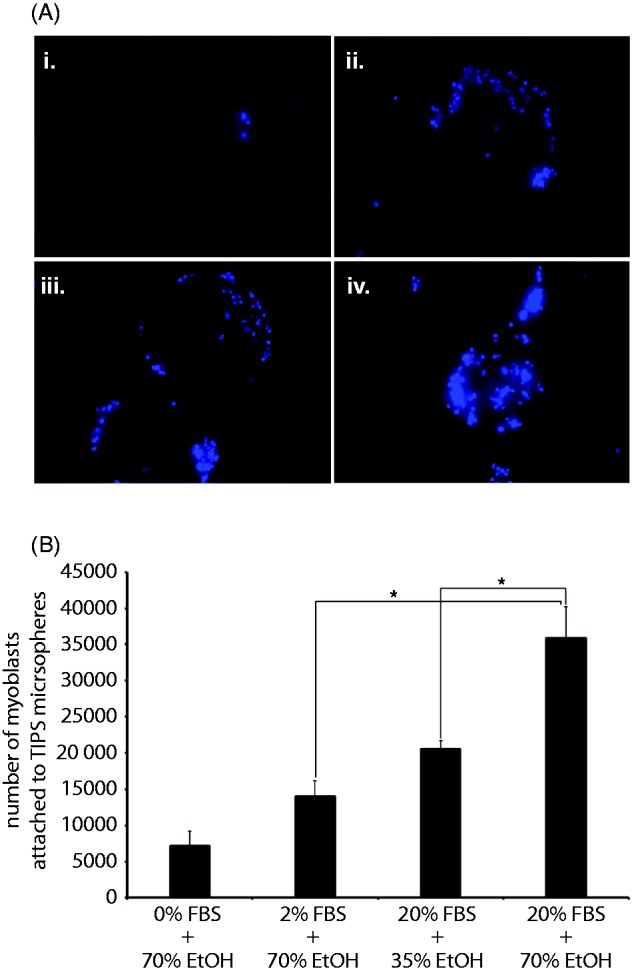
Human skeletal myoblast attachment to PLGA microspheres is dependent on the extent of pre-wetting and serum protein adsorption. Human skeletal myoblasts were subjected to dynamic culture (10 s shaking at approximately 400 r/min, every hour) for 6 h together with PLGA microspheres treated with 0% FBS-70% EtOH (Ai), 2% FBS-70% EtOH (Aii), 20% FBS-35% EtOH (Aiii) or 20% FBS-70% EtOH (Aiv) for 3 h. The nuclei of myoblasts attached to microspheres were stained with DAPI and imaged with excitation at 358 nm and emission at 461–488 nm. The Cyquant assay that measures DNA levels in cells was used to quantify the number of cells (B) attached to microspheres. Data points represent the mean (n = 3 ± SEM) number of cells. *p ≤ 0.05 indicate differences between cell attachment in various wetting conditions.

Cells have been shown to preferentially bind to wet surfaces^23^ and cell adhesion was reported to be dependent on the adsorption of serum proteins to substrates.^24^ We therefore investigated the attachment of human skeletal myoblasts to PLGA microspheres treated with 2% and 20% FBS, wetted with 35% and 70% ethanol (during a 3 h period) to determine conditions for optimal loading of these cells on the surface of the polymer scaffold. Myoblasts attached in greater numbers to microspheres wetted with 70% ethanol than those wetted with 35% ethanol regardless of a high serum protein concentration (20% FBS) (Figure 6). Cells were distributed on the peripheral regions of partially wetted microspheres (0% FBS + 70% ethanol (Ai), 2% FBS + 70% ethanol (Aii), 20% FBS + 35% ethanol (Aiii)) and they populated the whole as well as peripheral regions of the microsphere as wetness increased (20% FBS + 70% ethanol (Aiv)) (Figure 6a).

With lower amounts of serum protein (2% FBS) adsorbed to microspheres wetted with 70% ethanol, a significantly lower (*p* ≤ 0.05) number of cells attached than those treated with 20% FBS + 70% ethanol (Figure 6b). Greater numbers (*p* ≤ 0.05) of myoblasts attached to 20% FBS + 70% ethanol treated microspheres than those incubated with 20% FBS + 35% ethanol. Microspheres treated with 20% FBS + 35% ethanol allowed the attachment of similar numbers of cells as those treated with 2% FBS + 70% ethanol. In the absence of serum proteins and with 70% ethanol for wetting, five times less cells attached to microspheres than to those treated with 2% FBS pre-wetted with 70% ethanol. These data indicated that for skeletal myoblast loading to PLGA TIPS microspheres, wetting precedes the requirement for serum protein adsorption. Increasing the amount of serum proteins (ideally, autologous serum proteins) exposed to partially wet microspheres can, however, enhance cell loading.

**Figure 7.**
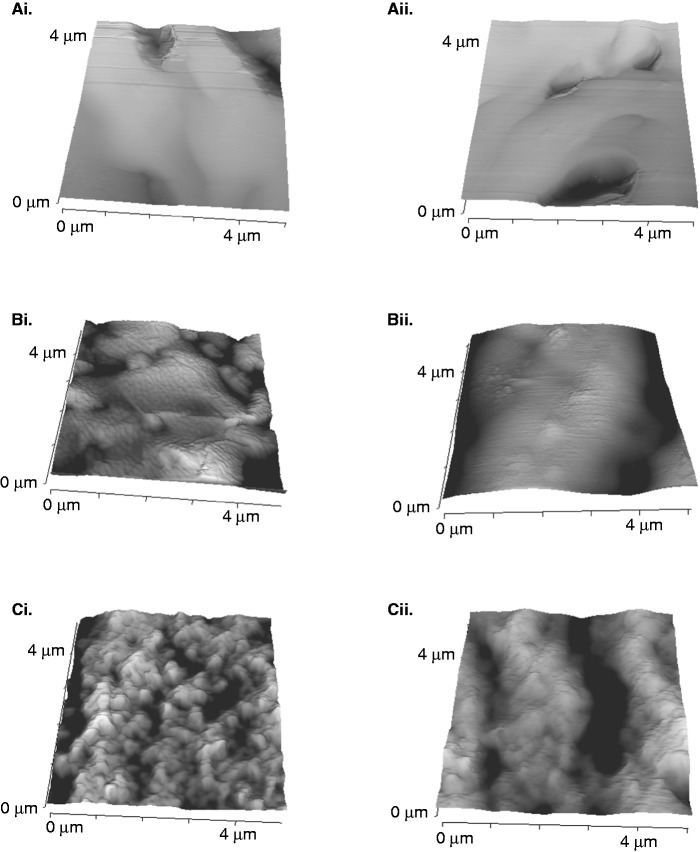
The surface topography of TIPS microspheres is modified by pre-wetting with ethanol. PLGA microspheres treated with 0% FBS and 0% ethanol (ai and aii) 20% FBS and 35% (v/v) (bi and bii) or 70% (v/v) (ci and cii) ethanol (EtOH) for 3 h were attached to metal stubs and imaged with a JPK NanoWizard II AFM mounted on an Olympus IX71 inverted microscope. Images of 5 × 5 µm dimensions were obtained at random sites on each sample. Images represent three individual experiments.

A hypothesis of the present study was that the differences observed in the numbers of cells attaching to microspheres may be due to changes in the surface topography due to variations in levels of wetting. Previous studies have shown that the addition of nano- and micro-features to PLGA microspheres increases endothelial cell attachment.^20^ Certainly, microspheres wetted with 70% (v/v) ethanol (Figure 7Ci and Cii) presented a more rugose surface structure than those treated with 35% (v/v) ethanol (Figure 7Bi and 7Bii), indicating that human skeletal myoblasts adhered more readily to a rough surface than a smooth surface (Figure 7).

The data described in the current study suggest that the surface topography of TIPS microspheres is an important modulator of cell attachment to these co-polymers that can be altered with ethanol-mediated wetting and serum protein adsorption to manipulate cell loading efficiency.

## Discussion

The clinical delivery of therapeutic cells could be significantly enhanced if they were delivered locally and retained in the tissue area requiring repair. Biocompatible PLGA microspheres were demonstrated to be wetted to various extents with ethanol and serum proteins via a simple method to control human skeletal myoblast loading. These cells may therefore be presented clinically on pre-wetted PLGA microspheres in varying amounts as necessary for specific treatment requirements.

In the present study, TIPS microspheres that are 4–9 times smaller (200–300 µm in diameter) than PLGA foam disks may be completely wetted with 100% ethanol after 24 h. Mikos et al.^3^ showed that 100% ethanol can wet PLGA foams in less than 24 h. This report demonstrated that wetting of PLGA foams in a two-step ethanol and water immersion method, allowed water entry into PLGA foam disks that were 1300–1730 µm in depth, and after only 1 h, water entry was close to its plateau value. The one-step ethanol immersion wetting procedure described in the present study may be suitable for applications requiring degradation of TIPS microspheres, for example, for the in vivo release of attached cells or encapsulated protein from this polymer. Wetting with ethanol in PBS has been shown to increase degradation of PLGA compared to wetting with PBS alone.^4^ Another advantage of the one-step method is that it is amenable to high-throughput production of pre-wetted PLGA microspheres that are safe and economically viable. TIPS microspheres were previously shown to present improved water wetting following treatment with atmospheric air plasma (AP: electrical ionization of gas) due to the formation of oxygen- and nitrogen-containing functions.^7^ Similar to ethanol-mediated wetting, AP can be applied in a production line setting for rapid treatment of TIPS microspheres, but this method presents safety and cost issues that are obviated by the one-step ethanol immersion protocol.

In line with findings described here, a previous study showed that endothelial adhesion strength is greater on hydrophilic positions on wettable polymers than on the hydrophobic positions.^11^ Interestingly, pre-adsorption of different serum proteins on polymers has been shown to cause differential cell adhesion – serum albumin prevented cell adhesion whereas the cell adhesion protein, fibronectin, enhanced cell adhesion independent of the extent of wetness.^25^ The adhesion of myoblasts to pre-wetted myoblasts in the present study may be due to competitive adsorption of cell adhesion proteins from the mixture of serum proteins.^19^ The microspheres prepared in the present study may only be moderately wetted, because studies have shown that cells attach to moderately hydrophilic surfaces and they do not attach to hydrophobic or completely hydrophilic surfaces.^11,12^

In the present study, only a proportion of the initial amount of serum protein exposed to TIPS microspheres were adsorbed, indicating that this material could be modified to improve protein adsorption. PLGA blended with Pluronic® F108,^18^ PEG^26,27^ or chitin^28^ were reported to adsorb greater amounts of protein than unmodified PLGA. Moreover, serum proteins adsorbed to TIPS microspheres may not change in structure, as serum albumin bound has been shown to retain its secondary structure and activity whilst bound to PLGA.^18^ Serum protein adsorption to PLGA is influenced by the method of manufacture and polymer composition. Differential binding of serum proteins to PLGA was described with albumin, IgG, transferring.^19^ Competitive adsorption of serum proteins from a mixture of albumin, IgG and fibronectin showed that the more abundant proteins albumin and IgG were adsorbed to polymers in greater amounts than fibronectin (important for cell adhesion but present at lower concentrations in plasma). In addition to influencing the extent of polymer wetness, protein coating of TIPS microspheres may be useful for *in vivo* transplantation. BSA- (BSA-NP) or transferrin (Tf-NP)-coated PLGA nanoparticles (NP) displayed a greatly prolonged half-life in blood when intravenously injected in rats and mice.^29^ *In vivo* targeting of healthy brain tissue was higher with Tf-NP than with BSA-NP and high numbers of both nanoparticles entered brain-developed tumors.

The influence of PLGA scaffold surface topography on cell attachment may be independent to the effects mediated by adsorbed serum protein. Nanostructured PLGA surfaces (cast from silastic molds of NaOH-treated nanostructured PLGA) supported the attachment of increased numbers of vascular cells (endothelial and aortic smooth muscle cells)^5^ and bladder smooth muscle cells compared with conventional, unmodified PLGA substrates.^20^ PLGA films with surfaces modified by solvent-mediated polymer casting on a master template (nanometer and micrometer features made using UV photolithography or electron beam lithography) were used to investigate the influence of surface topography on thrombogenic potential of PLGA.^21^ The study showed that submicron features increased adhesion of fibrinogen and platelets compared to unmodified, pristine PLGA films, so modification of the surface structure can reduce thrombogenicity of PLGA and render the polymer more suitable for use in blood vessels. PLGA membranes modified by oxygen and nitrogen plasma have also been shown to present increased surface rugosity that supported attachment of Vero cells.^6,30^

The extent of wetting mediated by one-step ethanol immersion and serum protein adsorption may benefit the development of PLGA TIPS microsphere scaffolds for improved clinical delivery of human skeletal myoblasts.

## Conclusions

**Figure 8.**
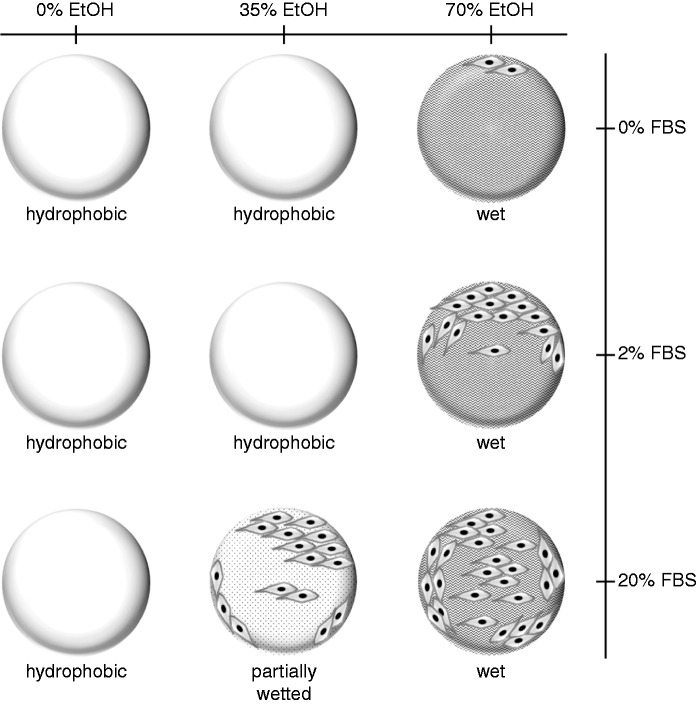
A working model of wetting conditions for attachment of human skeletal myoblasts to PLGA TIPS microspheres. PLGA TIPS microspheres incubated with various concentrations of ethanol (EtOH: 35%–70% (v/v)) and serum proteins (FBS:2–20%) allow the attachment of different numbers of skeletal myoblasts. The number of attached cells increase as the amount of serum protein exposed to microspheres is elevated. Microspheres that are partially wetted (35% EtOH) and incubated with high serum concentrations (20% FBS) allow attachment of similar numbers of cells as microspheres wetted (70% EtOH) under low-serum (2% FBS) conditions.

A simple one-step protocol has been described for pre-wetting PLGA TIPS microspheres that incorporates ethanol concentration and exposure time, serum protein adsorption and modifications to surface topography as critical parameters that direct the attachment of human skeletal myoblasts. The wetted TIPS microsphere presents a 3D myoblast culture system that can be tailored to control loading of these cells, indicating the possibility for regulation of the potency of skeletal myoblast therapy. We have devised a working model (Figure 8.) outlining the manner that wetting of TIPS microspheres can produce defined cell attachment profiles through variations to discrete elements of our pre-wetting procedure. The ultimate aim is to create a process for precision loading of human skeletal myoblasts onto PLGA microspheres. The present study is an important step in the routine clinical application of biocompatible PLGA TIPS microspheres. Wetted, serum protein-coated PLGA microspheres may ameliorate outstanding issues with the therapeutic efficacy of current myoblast transplantation procedures. Pre-wetted PLGA scaffolds are potentially tractable within high-throughput bioprocesses to create products for the delivery of a range of therapeutic, anchorage-dependent cell types including skeletal myoblasts, osteoblasts, chondrocytes and progenitor cells.
